# A New Biomarker Tool for Risk Stratification in “*de novo*” Acute Heart Failure (OROME)

**DOI:** 10.3389/fphys.2021.736245

**Published:** 2022-01-13

**Authors:** Rosa M. Agra-Bermejo, Carla Cacho-Antonio, Eva Gonzalez-Babarro, Adriana Rozados-Luis, Marinela Couselo-Seijas, Inés Gómez-Otero, Alfonso Varela-Román, José N López-Canoa, Isabel Gómez-Rodríguez, María Pata, Sonia Eiras, Jose R. González-Juanatey

**Affiliations:** ^1^Cardiovascular Area and Coronary Unit, University Clinical Hospital of Santiago de Compostela, Santiago de Compostela, Spain; ^2^Cardiology Group, Health Research Institute of Santiago de Compostela, Santiago de Compostela, Spain; ^3^CIBERCV: Centro de Investigación Biomédica en Red de Enfermedades Cardiovasculares, Madrid, Spain; ^4^Cardiovascular Area, Hospital Montecelo, Pontevedra, Spain; ^5^Traslational Cardiology Group, Health Research Institute of Santiago de Compostela, Santiago de Compostela, Spain; ^6^Biostatech, University of Santiago de Compostela, Santiago de Compostela, Spain

**Keywords:** inflammation, acute heart failure, adipokines, biomarkers, orosomucoid

## Abstract

**Background:** Inflammation is one of the mechanisms involved in heart failure (HF) pathophysiology. Thus, the acute phase reactant protein, orosomucoid, was associated with a worse post-discharge prognosis in *de novo* acute HF (AHF). However, the presence of anti-inflammatory adipokine, omentin, might protect and reduce the severity of the disease. We wanted to evaluate the value of omentin and orosomucoid combination for stratifying the risk of these patients.

**Methods and Results:** Two independent cohorts of patients admitted for *de novo* AHF in two centers were included in the study (*n* = 218). Orosomucoid and omentin circulating levels were determined by ELISA at discharge. Patients were followed-up for 317 (3–575) days. A predictive model was determined for the primary endpoint, death, and/or HF readmission. Differences in survival were evaluated using a Log-rank test. According to cut-off values of orosomucoid and omentin, patients were classified as UpDown (high orosomucoid and low omentin levels), equal (both proteins high or low), and DownUp (low orosomucoid and high omentin levels). The Kaplan Meier determined a worse prognosis for the UpDown group (Long-rank test *p* = 0.02). The predictive model that includes the combination of orosomucoid and omentin groups (OROME) + NT-proBNP values achieved a higher C-index = 0.84 than the predictive model with NT-proBNP (C-index = 0.80) or OROME (C-index = 0.79) or orosomucoid alone (C-index = 0.80).

**Conclusion:** The orosomucoid and omentin determination stratifies *de novo* AHF patients into the high, mild, and low risk of rehospitalization and/or death for HF. Its combination with NT-proBNP improves its predictive value in this group of patients.

## Introduction

Heart failure (HF) is the leading cause of hospitalization for patients > 65 years. In acute HF (AHF), exists a 20–30% of re-hospitalization within the first 3–6 months after discharge. It is coupled with an increase of acute-phase response reactants, measured by C-reactive protein (CRP) or interleukin-6 (IL-6) levels (Goonewardena et al., 2016) and a raise of other several serum inflammatory cytokines levels such as tumor necrosis factor alfa (TNF-α) and interleukin-1b (IL-1β) which are associated with the degree of disease severity, poor outcomes, and mortality (Goonewardena et al., 2016; Agra-Bermejo et al., 2020). A number of diagnostic and/or prognostic plasma biomarkers of worsening HF have been proposed during hospitalization and in chronic stable patients. Therefore, some examples of these biomarkers are: growth differentiation factor 15 (GDF15), that belongs to the transforming growth-factor beta superfamily and regulates inflammatory and apoptotic pathways; troponin T (TnT) that is a structural and contractile protein; ST2, a member of interleukin 1 receptor family, and galectin 3, a lectin family protein, that are involved in adverse cardiac remodeling and fibrosis; and TNF-a (Metra et al., 2007; Jankovic-Tomasevic et al., 2016; Boulogne et al., 2017). However, the European Society of Cardiology HF guidelines only recommend the determination of B-type natriuretic peptide (BNP) and N-terminal pro-brain natriuretic peptide (NT-proBNP) in clinical practice (Ponikowski et al., 2016). These proteins were considered biomarkers for evaluating dyspnoeic patients (Januzzi et al., 2005) and, in consequence, AHF. Moreover, they were included in the HF diagnostic algorithm and proposed for a more accurate follow-up of the patients and risk stratification. Several authors have suggested the combination of biomarkers associated with fibrosis (ST2 and Galectin 3) (Wang et al., 2016), inflammation (Ueland et al., 2015), or myocardial load/damage (BNP and TnI) (Behnes et al., 2015) as a better diagnostic and prognostic tool.

α-1-acid glycoprotein (AGP) or orosomucoid is an acute-phase protein mainly synthesized in the liver, but also in extrahepatic sites. While albumin is an acidic and neutral drug carrier, AGP is the primary carrier of basic (positively charged) drugs. The function of orosomucoid is still incompletely known, but it is believed that orosomucoid modulates the immune system in the acute-phase reaction. Inflammatory cytokines such as IL-1 and TNF-α can stimulate the production of orosomucoid by leukocytes (Fournier et al., 2000). Orosomucoid produced by endothelial cells has been shown to induce angiogenesis and enhance endothelial cell migration and capillary tube formation *in vitro*. Also, previous reports demonstrate that urinary levels of orosomucoid are increased in HF (Hou et al., 2014). Physiological orosomucoid levels improve the endothelial healing process (Fandiño-Vaquero et al., 2014). However, their high levels were associated with the pro-inflammatory profile of monocytes (Eiras et al., 2017). Thus, recent data from our group showed orosomucoid as a new prognosis biomarker in *de novo* AHF patients (Agra et al., 2017). Omentin is a novel adipokine preferentially produced by visceral adipose tissue with insulin-sensitizing effects, where circulating levels are decreased in insulin-resistant states; also, omentin causes vasodilatation of blood vessels and attenuates CRP-induced proinflammatory effect (Tan et al., 2010b). In consideration that omentin (a) is an anti-atherogenic and anti-inflammatory protein, (b) improves the protector effect of orosomucoid against lipotoxicity (Lage et al., 2015), and (c) is associated with better prognosis in patients with chronic stable HF (Narumi et al., 2014), our aim was to analyze the predictive prognosis value of the combined orosomucoid and omentin determination in acute *de novo* HF patients. We will get new biomarkers associated with HF progression in an earlier stage of the disease.

## Materials and Methods

### Study Population

This is a prospective observational study with two independent cohorts. One of the cohorts (DEXA) included 96 consecutive patients admitted at the Cardiology Department of Clinical Hospital of Santiago de Compostela between 2014 and 2015. The other cohort (SEC-ROVI) included 122 patients admitted at the Cardiology Department of Clinical Hospital of Santiago de Compostela and Pontevedra between 2018 and 2019. Heart failure diagnostic was made according to the recommendations of the European Society of Cardiology (Ponikowski et al., 2016).

The only exclusion criterion was the previous HF history. The database collected demographic, clinical (electrocardiogram and echocardiogram parameters within 24 h after admission) and laboratory analysis [haemogram, basic biochemistry and coagulation rate, lipid and glucose profile, as well as specialized parameters such as levels of electrolytes and pro-brain natriuretic peptide (NT-proBNP)], registered after admission.

The study complies with the Declaration of Helsinki and was approved by the Clinical Research Ethics Committee of Galicia. All patients provided informed consent.

### Orosomucoid and Omentin Plasma Levels Analysis

Blood samples were obtained at discharge and were centrifuged at 1,800 × g for 15 min. Isolated plasma was stored at –80°C until use. Orosomucoid levels from the DEXA cohort were analyzed by ELISA with a detection limit of 3,91 ng/mL (GenWay Biotech Inc.) (Agra et al., 2017) and from the SEC-ROVI cohort by ELISA with a detection limit of 59 ng/mL (SEA816Hu, Cloud Clone). Omentin levels were measured by ELISA with a detection limit of 6.5 pg/mL (SEA933Hu, Cloud Clone Corp, Houston, United States) following the protocol of the manufacturer.

NT-proBNP levels were determined at admission by the clinical laboratory according to the manufacturers’ protocol with a Roche reagent pack on an Elecsys 2010 immunoanalyzer (Roche Diagnostics) and European guidelines (McDonagh et al., 2021).

### Endpoint Definition and Follow-Up

The primary endpoint was death from any cause and/or HF rehospitalization. Follow-up information was recorded from medical history. The mean time of follow-up was 317 (3–575) days.

### Statistical Analysis

Continuous variables were represented by the mean and standard deviation (SD) or median and interquartile range if the distribution of the variables were not normal. Differences between the patients of each project were performed by ANOVA test or a Kruskall-Wallis test in the absence of normality. The categorical variables were represented with the absolute and relative frequencies and the differences between projects were evaluated using the χ^2^-test, or with the χ^2^-test with permutation if the expected frequency in any of the cells is less than 5. Differences in variables between patients who suffered or did not suffer an event (rehospitalization for HF and/or death) were determined by ANOVA test, or Kruskall-Wallis test, according to the normality of continuous variables. The differences on categorical variables were evaluated using the χ^2^-test, or with the χ^2^-test with permutation if the expected frequency in any of the cells was less than 5. For both continuous and categorical variables, the odds ratio (OR) was obtained by maximum likelihood estimation (MLE), and its 95% confidence interval (normal approximation) was represented. All survival analyzes were carried out using the Kaplan-Meier method. In addition, the differences in survival were evaluated based on each of the three categorized markers (according to the optimal cut-off point), using a Log-rank test. The optimal cut-off point for the markers was estimated with a methodology based on correlation with survival time: Maximally selected rank statistics. The cut-off point was the one that provides the most significant split based on the standardized log-rank test.

For the predictive models, several steps were performed:

(a) Analysis of missing values followed by multiple imputations of lost values with the MICE algorithm (Multiple Imputation by Chained Equations) (White et al., 2011). Fifteen imputations were made. The performance of imputation was evaluated by comparing the distributions of original and imputed values. Overimputation was used for evaluating the predictive distribution fitting. The pooling of estimated parameters was made following the rules of Rubin (1978) and Galbraith (2012).
(b) Analysis of the distribution of predictors to assess the presence of outliers.
(c) Possible transformations for each of the quantitative predictors. A univariate Cox model was estimated, and it was compared with predictor models with the logarithmic transformation, square root transformation, and restricted cubic splines. The presence of outliers followed a comparison with a model in which a truncation (winsoring) of the predictor had been carried out: a reasonable option was to choose the percentiles 1 and 99. Although only splines were applied to the markers.
(d) Categorical variables were included in the predictive model as a pathology score and a drug score. The scores were performed according to the Hazzard Ratio (HR) of the Univariant Cox models. The drug score considered (value 4 -nitrates intake; value 3-amiodarone; value 2-acid acetylsalicylic; value 1-the rest of drugs). The pathology score considered (value 3-hypertension; value 2-COPD, value 1.2-hyperlipidemia, value 1-rest of pathologies). To create these scales, a univariate Cox regression was carried out with each drug and pathologies, assigning a weight to each of the variables based on the HR obtained, and proceeding to add them to obtain the corresponding scores.
(e) Interactions with the markers and among them were tested by bivariate Cox models. The risk of proportionality was tested over time.
(f) The selection of the best predictive model was carried out with the LASSO method, and stepwise backward selection with validation through different resampling methods (bootstrap and randomization).
(g) An internal validation of the model was carried out using bootstrap.
(h) The discrimination capacity of the model (ability to distinguish event-free patients from those who present events) was estimated using the concordance index C, which is a generalization of the area under the curve (AUC). Time-dependent ROC curves were estimated.
(i) The model optimum was evaluated and corrected in the final parameters. Optimum is defined as the difference between the real model and the estimated one. For quantifying the added usefulness of the new biomarkers, the continuous Net Reclassification Index (NRI) and Integrated Discrimination Improvement (IDI) for survival models were estimated based on Pencina’s method (Pencina et al., 2008).
(j) The calibration of the model was carried out through bootstrap resampling, comparing the mean predicted by the model with the observed one.
(k) The prognostic models were presented in the form of a nomogram (which predicted survival at 3, 6, and 12 months), and using Kaplan-Meier curves with patients grouped according to the predicted risk profiles.

Regarding sample size estimation since the principal concern in clinical predictive modeling is the reduction of overfitting and precise estimation of parameters, we followed the sample size estimation method proposed by Riley et al. (2020). For estimating sample size with this methodology three criteria must be met: (a) small optimism (overfitting) in the estimation of model parameters which is reached by a shrinkage or penalization factor of 0.9 (the predictions in new patients will move away from the extremes and toward the mean), (b) reduce the difference between apparent and adjusted Nagelkerke’s \(R^2\) to a small value of 0.05, and (c) increase precision (\(\pm\) 0.05) in the estimation of the average risk in a selected time point in the target population. The required sample size corresponds with the one with the highest value among the three criteria. Required information is obtained from previous studies.

Furthermore, with the aim of minimizing problems regarding the overfitting due to a small sample size, regularization methods for selecting candidate predictors were used as well as internal validation to reduce the optimism in estimation.

The statistical analysis was performed by R software. The packages mice (Rubin, 1978; van Buuren, 2007; Groothuis-oudshoorn, 2011; Galbraith, 2012), miceadds (Robitzsch and Grund, 2020), and Hmisc (Harrell and Frank, 2018) were used for imputation and pooling; timeROC (Blanche et al., 2013) for obtaining time-dependent ROC curves; glmpath (Park and Hastie, 2018) for model selection, and rms (Harrell and Frank, 2018) for model selection, validation, calibration, and prognostic models presentation.

## Results

### Baseline Characteristics of Patients

After missing some clinical values in one of the two cohorts from 17 patients, we included the main clinical characteristics of 201 patients for their comparison (Supplementary Table 1). The mean age was 71.0 (61.0;79.0) years old with a BMI of 29.0 (26.8;33.6) kg/m^2^. Of all patients, 65.7% were men, 39.6% drank alcohol, 22.2% were smokers, 35.3% were diabetic, 68.2% were hypertensive, 47% had edemas at admission, and 12.9%, chronic obstructive pulmonary disease (COPD). The main differences between the two cohorts (DEXA and SEC-ROVI) were alcohol intake, BMI, percentage of patients with edemas at admission, hepatomegaly, and previous stroke.

### Quantile Levels of Orosomucoid and Omentin

Orosomucoid levels were measured in 160 patients stratified in 3 quantiles (Q1, the lowest levels, Q2 and Q3, the highest levels). Those patients with highest orosomucoid levels (Q3 or Q2) had higher glucose levels 130 (98.2;177) or 163 (121;243) mg/dL, respectively, than those patients in Q1 122 (106;183], *p* = 0.008. The highest levels of creatinine were described in Q3 patients 1.21 (0.86;1.47) vs. 0.95 (0.77;1.10) in Q1 or 0.87 (0.75;1.02) mg/dL in Q2, *p* = 0.001. The percentage of patients with an event was higher in the Q3 group 16 (31.4%) vs. 6 (10.3%) in Q1 or 9 (15.0%) in Q2, *p* = 0.013 (Table 1). Regarding omentin, which was measured in 155 patients, a higher percentage of smokers and alcohol intake was represented on the Q1 group (lowest omentin levels) (Table 2). The ischemic etiology was represented in a similar percentage among groups of orosomucoid or omentin quantiles. The orosomucoid Q1, Q2, and Q3 were represented by 16, 19, and 25%; *p* = 0.458 and the omentin Q1, Q2, and Q3 by 18, 24, and 20%; *p* = 0.774, respectively.

**TABLE 1:** Clinical characteristics differences among orosomucoid quantiles groups.

Orosomucoid (mg/mL)	Q1 (0.82 ± 0.18)	Q2 (1.27 ± 0.15)	Q3 (2.40 ± 0.54)	P.overall
	*N* = *52*	*N* = *57*	*N* = *51*	
Sex (Women)	19 (36.5%)	20 (35.1%)	17 (33.3%)	0.943
Age	70.5 [61.0;79.2]	70.0 [62.0;78.0]	73.0 [60.0;78.0]	0.988
Alcohol	18 (36.0%)	26 (46.4%)	13 (25.5%)	0.080
Tobacco	10 (20.0%)	19 (33.3%)	7 (13.7%)	**0.045**
BMI	30.4 [27.6;35.7]	28.9 [26.6;33.6]	28.5 [26.6;32.5]	0.292
Previous peripheral artery	5 (9.62%)	3 (5.26%)	5 (9.80%)	0.642
Ascites	0 (0.00%)	0 (0.00%)	1 (2.00%)	1.000
DM	19 (36.5%)	21 (36.8%)	17 (33.3%)	0.917
Edemas_admission	27 (51.9%)	25 (43.9%)	29 (56.9%)	0.392
COPD	7 (13.5%)	7 (12.3%)	7 (13.7%)	0.972
Hepatomegalia	2 (4.35%)	5 (10.4%)	9 (18.0%)	0.102
HLP	29 (55.8%)	29 (50.9%)	29 (56.9%)	0.799
HTA	37 (71.2%)	36 (63.2%)	38 (74.5%)	0.417
Previous myocardial infarction	4 (7.69%)	7 (12.3%)	8 (15.7%)	0.452
Previous Stroke	3 (5.77%)	3 (5.26%)	3 (5.88%)	1.000
Heart rate	69.2 (14.2)	72.2 (13.1)	69.6 (12.8)	0.465
Systolic blood preasure_admission	141 (27.3)	139 (27.1)	143 (26.7)	0.745
Salicylic acid	13 (25.0%)	21 (38.2%)	17 (33.3%)	0.339
Amiodarone	5 (9.62%)	5 (9.09%)	10 (19.6%)	0.192
ARB	12 (23.1%)	6 (10.9%)	14 (27.5%)	0.088
Betablockers	39 (75.0%)	47 (85.5%)	39 (76.5%)	0.352
Digoxin	15 (28.8%)	11 (20.0%)	11 (22.0%)	0.532
Diuretics	48 (92.3%)	51 (92.7%)	49 (96.1%)	0.780
ACEI	33 (63.5%)	40 (72.7%)	28 (54.9%)	0.161
Ivabradine	2 (3.85%)	4 (7.27%)	3 (5.88%)	0.836
Metformin	15 (28.8%)	22 (40.0%)	9 (17.6%)	0.041
Nitrates	3 (5.77%)	3 (5.45%)	3 (5.88%)	1.000
Creatinine_admission (mg/dL)	0.95 [0.77;1.10]	0.87 [0.75;1.02]	1.21 [0.86;1.47]	**0.001**
LVEF_admission				0.756
< 40%	18 (40.0%)	29 (51.8%)	20 (48.8%)	
40–49%	11 (24.4%)	9 (16.1%)	7 (17.1%)	
> 50%	16 (35.6%)	18 (32.1%)	14 (34.1%)	
Glucose_admission (mg/dL)	122 [106;183]	163 [121;243]	130 [98.2;177]	**0.008**
HB_admission (g/dL)	13.5 (2.16)	13.7 (1.93)	13.4 (1.85)	0.673
K_admission (mmol/L)	4.41 (0.55)	4.38 (0.52)	4.60 (0.68)	0.108
Na_admission (mmol/L)	140 [139;143]	140 [138;142]	141 [138;144]	0.232
NT-ProBNP_admission (pg/mL)	2,297 [1,180;3,518]	2,064 [916;4,380]	3,645 [1,372;7,324]	0.111

*p-values lower than 0.05 are in bold.*

**TABLE 2:** Clinical characteristics differences among omentin quantiles groups.

Omentin (ng/mL)	Q1 (3.75 ± 1.07)	Q2 (6.87 ± 0.78)	Q3 (10.82 ± 2.43)	P.overall
	*N* = 51	*N* = 55	*N* = 49	
Sex (Women)	16 (31.4%)	16 (29.1%)	22 (44.9%)	0.196
Age	72.0 [62.5;79.5]	68.0 [60.0;78.0]	68.0 [61.0;79.0]	0.269
Alcohol	26 (54.2%)	20 (36.4%)	10 (20.4%)	**0.003**
Tobacco	16 (32.7%)	14 (25.5%)	5 (10.2%)	**0.026**
BMI	30.4 [27.6;33.9]	29.2 [26.5;33.9]	28.8 [27.6;33.6]	0.621
Previous peripheral artery	5 (9.80%)	5 (9.09%)	3 (6.12%)	0.828
Ascites	0 (0.00%)	1 (1.92%)	0 (0.00%)	1.000
DM	18 (35.3%)	19 (34.5%)	20 (40.8%)	0.775
Edemas_admission	25 (49.0%)	25 (45.5%)	29 (59.2%)	0.355
COPD	9 (17.6%)	6 (10.9%)	5 (10.2%)	0.465
Hepatomegalia	4 (10.0%)	6 (11.5%)	5 (10.6%)	1.000
HLP	24 (47.1%)	31 (56.4%)	29 (59.2%)	0.440
HTA	30 (58.8%)	41 (74.5%)	38 (77.6%)	0.085
Previous myocardial infarction	4 (7.84%)	10 (18.2%)	5 (10.2%)	0.233
Previous Stroke	3 (5.88%)	2 (3.64%)	3 (6.12%)	0.827
Heart rate	72.6 (12.2)	67.4 (14.5)	71.7 (12.4)	0.108
Systolic blood pressure	139 (24.7)	137 (26.7)	146 (27.3)	0.169
Salicylic Acid	16 (32.0%)	21 (38.2%)	12 (25.0%)	0.360
Amiodarone	4 (8.00%)	10 (18.2%)	3 (6.25%)	0.110
ARB	8 (16.0%)	11 (20.0%)	12 (25.0%)	0.540
Betablockers	36 (72.0%)	47 (85.5%)	39 (81.2%)	0.219
Digoxin	8 (16.0%)	11 (20.0%)	17 (36.2%)	**0.047**
Diuretics	45 (90.0%)	53 (96.4%)	45 (93.8%)	0.471
ACEI	32 (64.0%)	36 (65.5%)	31 (64.6%)	0.988
Ivabradine	3 (6.00%)	3 (5.45%)	3 (6.25%)	1.000
Metformin	18 (36.0%)	14 (25.5%)	12 (25.0%)	0.386
Nitrates	2 (4.00%)	6 (10.9%)	1 (2.08%)	0.155
Creatinine_admission (mg/dL)	1.03 [0.85;1.27]	0.97 [0.80;1.27]	0.88 [0.72;1.08]	0.069
LVEF_admission				0.602
< 40%	22 (46.8%)	24 (50.0%)	19 (45.2%)	
40–49%	6 (12.8%)	11 (22.9%)	8 (19.0%)	
> 50%	19 (40.4%)	13 (27.1%)	15 (35.7%)	
Glucose_admission (mg/dL)	136 [113;214]	130 [100;189]	152 [107;190]	0.325
HB_admission (g/dL)	13.3 (2.28)	13.6 (1.88)	13.6 (1.79)	0.767
K_admission (mmol/L)	4.52 (0.55)	4.45 (0.54)	4.41 (0.70)	0.621
Na_admission (mmol/L)	140 [138;142]	140 [138;143]	141 [139;143]	0.179
NT-ProBNP_admission (pg/mL)	2,242 [1,186;4,669]	2,440 [908;4,408]	2,202 [1,128;4,784]	0.925

*p-values lower than 0.05 are in bold.*

### Marker’s Correlation

The correlation among markers showed that neither orosomucoid nor omentin showed statistically significant correlations with any clinical variable (Supplementary Figure 1).

### Cut-Off Values of Orosomucoid and Omentin

We performed the imputation of the lost values using the MICE algorithm (Multiple imputation by chained equations). Confidence intervals obtained by overimputation showed a great degree of coverage for all the variables (for all the imputed variables, the overimputed confidence interval included the observed values in more than 90% of the cases). The distribution of imputed and original values was very close. Afterward, the median was defined as the optimal cut-off point, threshold to consider high or low values of the markers, for orosomucoid and omentin. The omentin and orosomucoid cut-off values were 6.637 and 1.409, respectively. Then, the following categories were generated: (a) DownUp: Orosomucoid values below optimal and omentin values above optimal, (b) Equal: both proteins were simultaneously above the optimum, or below it, (c) UpDown: orosomucoid values above optimal and omentin values? below optimal. The main differences among groups were the higher percentage of Chronic obstructive pulmonary disease (COPD), creatinine and potassium levels at admission in the UpDown group (Table 3).

**TABLE 3:** Clinical characteristics differences among categorized (OROME) groups.

Orosomucoid/ Omentin (OROME)	DownUp	Equal	UpDown	P.overall
	*N* = *43*	*N* = *89*	*N* = *22*	
Sex (Women)	21 (48.8%)	27 (30.3%)	6 (27.3%)	0.080
Age	67.0 [59.5;79.0]	71.0 [61.0;78.0]	72.5 [62.8;78.0]	0.652
Alcohol	13 (30.2%)	35 (40.7%)	8 (36.4%)	0.509
Tobacco	6 (14.0%)	25 (28.7%)	4 (18.2%)	0.143
BMI	28.9 [27.4;35.1]	29.9 [27.1;32.8]	28.6 [26.4;34.1]	0.987
Previous peripheral artery	2 (4.65%)	8 (8.99%)	2 (9.09%)	0.696
DM	16 (37.2%)	35 (39.3%)	5 (22.7%)	0.347
Edemas_admission	25 (58.1%)	43 (48.3%)	11 (50.0%)	0.566
COPD	1 (2.33%)	15 (16.9%)	4 (18.2%)	**0.041**
Hepatomegalia	2 (4.76%)	9 (12.2%)	4 (18.2%)	0.236
HLP	24 (55.8%)	50 (56.2%)	9 (40.9%)	0.418
HTA	34 (79.1%)	57 (64.0%)	17 (77.3%)	0.153
Previous myocardial infarction	5 (11.6%)	11 (12.4%)	3 (13.6%)	1.000
Previous Stroke	3 (6.98%)	4 (4.49%)	1 (4.55%)	0.873
Heart rate	69.7 (14.1)	71.3 (13.2)	68.4 (12.4)	0.628
Systolic blood preasure_admission	144 (28.3)	139 (26.4)	140 (23.6)	0.555
Salicylic acid	13 (30.2%)	28 (32.2%)	7 (31.8%)	0.975
Amiodarone	4 (9.30%)	9 (10.3%)	4 (18.2%)	0.601
ARB	8 (18.6%)	20 (23.0%)	3 (13.6%)	0.595
Betablockers	38 (88.4%)	67 (77.0%)	16 (72.7%)	0.210
Digoxin	14 (32.6%)	18 (20.9%)	4 (18.2%)	0.274
Diuretics	41 (95.3%)	79 (90.8%)	22 (100%)	0.253
ACEI	31 (72.1%)	54 (62.1%)	13 (59.1%)	0.452
Ivabradine	1 (2.33%)	7 (8.05%)	1 (4.55%)	0.458
Metformin	12 (27.9%)	28 (32.2%)	4 (18.2%)	0.426
Nitrates	3 (6.98%)	4 (4.60%)	2 (9.09%)	0.715
Creatinine_admission (mg/dL)	0.84 [0.70;1.00]	1.00 [0.82;1.28]	1.21 [0.94;1.30]	**0.001**
LVEF_admission				0.918
< 40%	17 (43.6%)	41 (51.2%)	7 (41.2%)	
40–49%	8 (20.5%)	14 (17.5%)	3 (17.6%)	
> 50%	14 (35.9%)	25 (31.2%)	7 (41.2%)	
Glucose_admission (mg/dL)	127 [107;192]	137 [108;193]	132 [111;189]	0.919
HB_admission (g/dL)	13.6 (1.91)	13.5 (2.08)	13.2 (1.80)	0.785
K_admission (mmol/L)	4.25 (0.54)	4.52 (0.62)	4.63 (0.46)	**0.017**
Na_admission (mmol/L)	140 [139;143]	140 [138;143]	142 [139;144]	0.192
NT-ProBNP_admission	2,360 [1,110;3,815]	2,086 [1,126;5,261]	3,431 [1,052;5,368]	0.677

*p-values lower than 0.05 are in bold.*

### Follow-Up and Events

**FIGURE 1:**
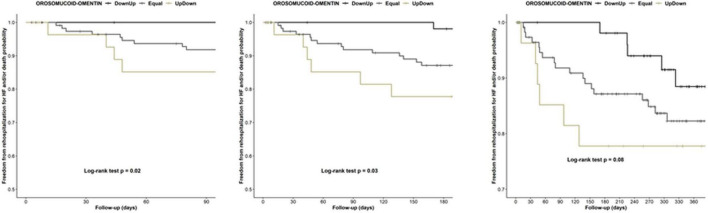
Kaplan Meier curves display the rehospitalization for HF and/or death probability for the three categorized groups (DownUp-low orosomucoid and high omentin, Equal-both protein low or high, UpDown-high oromucoid and low omentin levels) at 3, 6, and 12 months after discharge.

During the follow-up, 16% of patients were rehospitalized for HF and/or death. These patients were older, mostly hypertensive, with high NT-proBNP and high creatinine levels at admission with respect to those without events (Supplementary Table 2). Patients in the UpDown group had the worse prognosis at 90, 180, and 365 days. The Kaplan-Meier curves showed that these differences were statistically significant (*p* < 0.05) (Figure 1).

After testing the number of events in the NT-proBNP, orosomucoid, and omentin categorized groups by quantiles and the combined orosomucoid and omentin group regarding cut-off values (Up or down), we observed that all the events in the DownUp group, low orosomucoid, and high omentin levels, were recorded 6 months after discharge (Supplementary Figure 2). Contrary, the UpDown group had events in the following 30 days after discharge.

### Predictive Models for Rehospitalization for Heart Failure and/or Death

**FIGURE 2:**
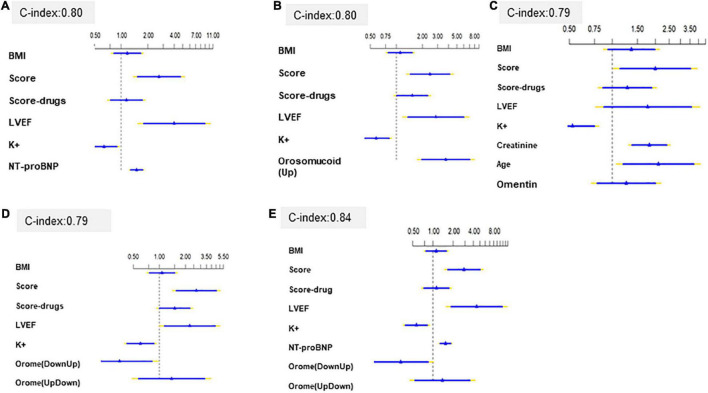
Predictive models with **(A)** NT-proBNP, **(B)** Orosomucoid, **(C)** Omentin, **(D)** OROME, **(E)** NT-proBNP and OROME and hazard ratio and 95% confidence interval for events of each variable.

Pathologies were grouped in a Score, assigning 3 points if the patient had hypertension, 2 points if they have COPD, 1.2 points if they have hyperlipidemia, and 1 point to the remaining pathologies. For the treatments, the variable Pharmacological-score was generated, assigning 4 points if the patient takes nitrates, 3 points if the patient takes amiodarone, 2 points if the patient takes acetylsalicylic acid, and 1 point to the other drugs. Three different methodologies, the Lasso method and Backward Step-down deletion with and without bootstrapped validation (200 bootstrap samples) with AIC as a stopping rule, were performed for selecting the variables for the best predictive model. Among the most frequently selected covariates were LVEF, K^+^, Creatinine, BMI, and pharmacological and pathology scores. NT-ProBNP was selected in every model. With regard to Omentin and Orosomucoid, their selection was increased when their interaction with any of the scores was also included (Supplementary Table 3). After testing the predictive models that include the same clinical variables of BMI, pathology and pharmacological scores, LVEF and K^+,^ and the biomarkers, we observe that the inclusion of NT-proBNP determined a c-index = 0.8016 (optimism corrected C-index = 0.798) (Central Illustration), the inclusion of orosomucoid depicted a c-index = 0.8036 (optimism corrected C-index = 0.788) (Figure 2), the inclusion of ometin a c-index = 0.7953 (optimism corrected C-index = 0.772) and the combination of categorized orosomucoid and omentin (UpDown, Equal or DownUp) (OROME) had similar c-index = 0.7889 (optimism corrected C-index = 0.778) (Figure 2). But the inclusion of NT-proBNP and the combined orosomucoid and omentin (OROME) showed the best c-index = 0.8488 (Figure 2). These results indicated the improvement of the predictive model with all three biomarkers (Figure 2).

The AUC were sustained in the time (Supplementary Figure 3A) with model OROME + NT-proPNB outperformed model NT proBNP (Supplementary Figure 3B). Models including only combination of orosumucoid and omentin (OROME) or orosomucoid performed as well as NT proBNP model (Supplementary Figures 3C,D).

The additional value of the new biomarker according NRI is 31.2% (95%CI = 0.01–54.8%, *p*-value = 0.05), with and IDI of 4.9% (95% CI = 0.1–12.1%, *p*-value = 0.04).

Calibration plots showed very good concordance between predicted and observed survival probabilities for the selected model (Supplementary Figure 4).

**FIGURE 3:**
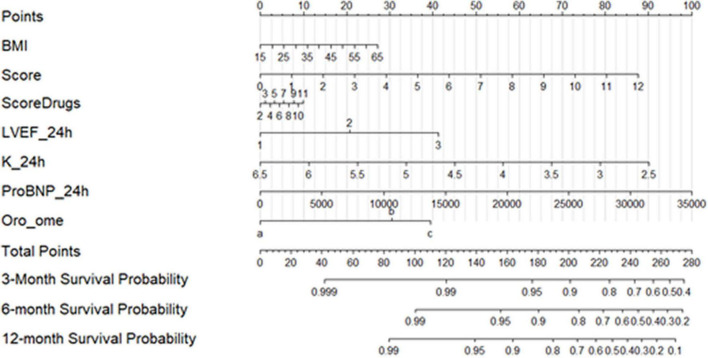
Nomogram of finally selected model (including BMI, pathology and pharmacological scores, LVEF, K+, NT-proBNP, and combination of orosomucoid and omentin).

Nomogram of the finally selected model (including BMI, pathology and pharmacological scores, LVEF, K^+^, NT-proBNP, and OROME) is presented in Figure 3. It shows predicted survival probabilities at several follow-up times. The total points are obtained by adding the individual points of each predictor. These points are obtained by drawing a vertical straight line from the predictor value to Points (i.e., 42.5 points are assigned to a concentration of NT-ProBNP of 15,000 ng/mL).

## Discussion

Our results show that the combination of plasma orosomucoid and omentin values improved the prognostic risk stratification of *de novo* AHF patients. Therefore, low orosomucoid and high omentin levels were associated with the lowest risk of suffering adverse outcomes during the follow-up compared with UpDown or equal group (high orosomucoid and low omentin levels or both proteins high or low). Adding these two new biomarkers to NT-ProBNP significantly improves the risk stratification of acute *de novo* HF patients.

To the best of our knowledge, we describe for the first time the prognostic value of these biomarkers in *de novo* AHF patients. Our findings may have implications not only for the risk stratification at hospital discharge because these new biomarkers can be considered as a potential therapeutic target and useful for the development of new treatment strategies and help to improve the patient care pathway.

Nowadays *de novo* AHF represent one-third of hospitalized patients for AHF (Hummel et al., 2015). Although their mortality in hospital is similar to patients with worsening HF, they have better post-discharge follow-up, fewer associated comorbidities, and less advanced HF syndrome (Banno et al., 2011; Lassus et al., 2011). However, their long-term prognosis after hospitalization for HF remains poor. Our data showed that 16% of patients had an event during the 1-year mean follow-up. Thus, many efforts are focused on these patients with the aim of achieving hemodynamic stability, preventing recurrent HF, and reducing mortality (Agra et al., 2017).

The present study shows that admitted patients for *de novo* AHF with high orosomucoid and low omentin levels presented the highest risk of death or requiring re-hospitalization for HF during an early or mid-term follow-up. However, any patients with low plasma orosomucoid and high omentin levels had an event within 100 days after discharge. These patients represent 40% of the *de novo* AHF patients. Therefore, there is an improvement in risk stratification of patients with *de novo* AHF using the combination of two proteins (high concentration of orosomucoid and low concentration of omentin).

One of the main issues, in AHF management, is based on the identification of patients with worse prognoses for improving their management and trying to find optimal medical treatments and an efficient follow-up plan. Some clinical parameters (heart rate at the admission (Lourenço et al., 2016), blood pressure, renal failure (Hummel et al., 2015) or protein biomarkers [NT-proBNP (Fonarow et al., 2007), ST2 (Shah and Januzzi, 2010), galectin-3 (Lainscak et al., 2010)] or other scores were used to this end. However, all of them had limitations. Recent data showed that a cardiac-specific marker, DKK3, also had a limited additional prognostic value regarding NT-proBNP (Piek et al., 2020). A multimarker panel was suggested in patients with AHF and renal mild or moderate impairment or the combination of two biomarkers in dyspnoeic patients. However, this is the first time that a protective and a deleterious marker combination were tested in *de novo* AHF for stratifying patients according to mortality risk or readmission for HF. The combination of NT-proBNP, orosomucoid, and omentin took out the best predictive model for rehospitalization for HF and/or death. A nomogram strategy might change the clinical follow-up of the patients (Figure 3).

The pathogenesis of *de novo* HF is not completely known and an inflammatory mechanism has been suggested (Goonewardena et al., 2016). The association between inflammation and HF has been recognized after many studies demonstrated that pro-inflammatory biomarkers are elevated in patients with a variety of cardiomyopathies and the clinical presentation of the HF syndrome. The plasma levels of inflammatory biomarkers have correlated with the prognosis and severity of the disease in both reduced and preserved LVEF-HF patients (Lainscak et al., 2010; Banno et al., 2011; Lassus et al., 2011). Moreover, higher inflammatory cytokines were associated with chronic kidney disease and a worse prognostic in patients with AHF (Pugliese et al., 2020). Although, our population debuted with the disease and creatine levels were in a normal range, the inflammatory pathway might also add value at the first stages of the disease.

In this sense, low levels of omentin, an anti-inflammatory cytokine, have been described in chronic HF patients (Narumi et al., 2014) and higher levels of orosomucoid have been related to worse outcomes in acute decompensated heart failure patients (Agra et al., 2017). These proteins have a protective effect on cardiomyoblasts, in part, mediated by an anti-lipotoxicity effect (Lage et al., 2015).

Omentin might play a cardioprotective role against an inflammatory process and high orosomucoid levels since its high levels are able to reduce the probability of death or readmission for HF in those patients with high orosomucoid levels.

The role of our discovery in the pathogenesis and prognosis of HF has potential therapeutic and diagnostic implications. We could not prove if these proteins are a direct cause of HF, but if it is confirmed in further studies, the treatment targeting this inflammatory pathway should be beneficial; however, if it is only a marker of the disease it could still help us to identify patients who are in a more advanced state in the moment of the diagnosis and improve the following-up of patients with a higher risk of a new event. Some studies were not able to demonstrate that physical activity might improve the omentin levels in a rat obese model (Costa et al., 2021). Other authors have suggested a benefit from metformin in women with polycystic ovary syndrome (Tan et al., 2010a). However, we did not find differences among groups regarding diabetes treatment or presence. The findings of this manuscript complement recent findings around the mechanisms of the benefits of SGLT2i in HF (Requena-Ibáñez et al., 2021). In this article, SGLT2i both reduce epicardial adipose tissue (which would result in increased omentin levels) and cause an anti-inflammatory effect (which would be translated as lower levels of orosomucoid). Therefore, the OROME score perfectly captures the benefits of SGLT2i in HF (Santos-Gallego et al., 2021) given that higher levels of omentin and lower levels of orosomucoid both predict improved outcomes (as per the OROME score) and are also a consequence of SGLT2i treatment (as demonstrated in Requena-Ibáñez et al. (2021).

### Limitations

The number of patients included is small but is comparable to other studies that assessed similar objectives and outcomes and the results are statistically significant and we think that this issue will have future clinical relevance. The adjustment for other clinical parameters and biomarkers may influence our results.

## Conclusion

The combination of orosomucoid, omentin, and NT-proBNP could be a new biomarker strategy for risk stratification in patients with *de novo* HF. Our findings should be considered as hypothesis-generating and need to be confirmed in future large-scale cohorts of patients with the spectrum of HF clinical presentation.

## Data Availability Statement

The original contributions presented in the study are included in the article/Supplementary Material, further inquiries can be directed to the corresponding author/s.

## Ethics Statement

The studies involving human participants were reviewed and approved by the Galician Clinical Committee. The patients/participants provided their written informed consent to participate in this study.

## Author Contributions

RA-B, CC-A, EG-B, JL-C, IG-O, AV-R, IG-R, and JG-J included the patients and clinical data. MC-S, AR-L, and SE included the laboratory measures. RA-B, SE, and JG-J participated in the design of the study. MP performed statistical analysis. All authors wrote the manuscript.

## Conflict of Interest

The authors declare that the research was conducted in the absence of any commercial or financial relationships that could be construed as a potential conflict of interest.

## Publisher’s Note

All claims expressed in this article are solely those of the authors and do not necessarily represent those of their affiliated organizations, or those of the publisher, the editors and the reviewers. Any product that may be evaluated in this article, or claim that may be made by its manufacturer, is not guaranteed or endorsed by the publisher.

## Abbreviations

AHF: acute heart failure
HF: heart failure
LVEF: left ventricular ejection fraction
NT-proBNP: N-terminal pro-brain natriuretic peptide.
