# Supplementary material for: A New Biomarker Tool for Risk Stratification in “de novo” Acute Heart Failure (OROME)
Source: Front Physiol. 2022 Jan 13;12:736245. doi: 10.3389/fphys.2021.736245 (PMC8793744; doi:10.3389/fphys.2021.736245)
Supplement: Supplementary file 1 [file Table_1.docx]

**Suplemmentary Table 1. Clinical characteristics differences between two included cohorts**

|  | **[ALL]** | **DEXA** | **SEC_ROVI** | **p.overall** |
| --- | --- | --- | --- | --- |
|  | *N=201* | *N=96* | *N=105* |  |
| **Sex (Women)** | 69 (34.3%) | 37 (38.5%) | 32 (30.5%) | 0.292 |
| **Age** | 71.0 [61.0;79.0] | 69.0 [61.0;78.0] | 72.0 [62.0;80.0] | 0.233 |
| **Alcohol** | 78 (39.6%) | 22 (22.9%) | 56 (55.4%) | **<0.001** |
| **Tobacco** | 44 (22.2%) | 16 (16.7%) | 28 (27.5%) | 0.098 |
| **BMI (kg/m2)** | **29.0 [26.8;33.6]** | **30.8 [27.7;34.8]** | **28.5 [26.2;32.0]** | **0.016** |
| **Previous peripheral artery** | 22 (10.9%) | 8 (8.33%) | 14 (13.3%) | 0.364 |
| **Ascites** | 2 (1.08%) | 1 (1.04%) | 1 (1.11%) | 1.000 |
| **DM** | 71 (35.3%) | 35 (36.5%) | 36 (34.3%) | 0.862 |
| **Edemas_admission** | 94 (47.0%) | 59 (61.5%) | 35 (33.7%) | **<0.001** |
| **COPD** | 26 (12.9%) | 11 (11.5%) | 15 (14.3%) | 0.699 |
| **Hepatomegalia** | 17 (9.60%) | 14 (14.6%) | 3 (3.70%) | **0.028** |
| **HLP:** | 107 (53.2%) | 53 (55.2%) | 54 (51.4%) | 0.693 |
| **HTA** | 137 (68.2%) | 70 (72.9%) | 67 (63.8%) | 0.218 |
| **Previous myocardial infarction** | 21 (10.4%) | 12 (12.5%) | 9 (8.57%) | 0.497 |
| **Previous Stroke** | 9 (4.48%) | 8 (8.33%) | 1 (0.95%) | **0.010** |
| **Atrial fibrillation** | 80 (40%) | 38(39.5%) | 42(40%) | 0.672 |
| **Heart rate** | 71.5 [60.0;80.0] | 70.5 [59.0;77.2] | 75.5 [61.0;81.0] | 0.053 |
| **Systolic blood preasure** | 141 (27.1) | 140 (27.8) | 142 (26.5) | 0.520 |
| **Salicylic Acid** | 58 (29.6%) | 29 (30.2%) | 29 (29.0%) | 0.977 |
| **Amiodarone** | 21 (10.7%) | 16 (16.7%) | 5 (4.95%) | **0.015** |
| **ARB** | 39 (19.8%) | 23 (24.0%) | 16 (15.8%) | 0.211 |
| **Betablockers** | 159 (80.7%) | 77 (80.2%) | 82 (81.2%) | 1.000 |
| **Digoxin** | 40 (20.4%) | 27 (28.4%) | 13 (12.9%) | **0.012** |
| **Diuretics** | 186 (94.4%) | 93 (96.9%) | 93 (92.1%) | 0.248 |
| **Hidralazine** | 1 (0.51%) | 0 (0.00%) | 1 (0.99%) | 1.000 |
| **ACEI** | 125 (63.5%) | 66 (68.8%) | 59 (58.4%) | 0.175 |
| **Ivabradine** | 9 (4.57%) | 4 (4.17%) | 5 (4.95%) | 1.000 |
| **Metformin** | 56 (28.4%) | 22 (22.9%) | 34 (33.7%) | 0.130 |
| **Nitrates** | 12 (6.09%) | 4 (4.17%) | 8 (7.92%) | 0.422 |
| **Creatinine_admission (mg/dL)** | 0.97 [0.80;1.22] | 0.98 [0.78;1.22] | 0.96 [0.82;1.23] | 0.671 |
| **LVEF_admission** |  |  |  | 0.447 |
| <40% | 91 (50.0%) | 37 (47.4%) | 54 (51.9%) |  |
| 40-49% | 30 (16.5%) | 16 (20.5%) | 14 (13.5%) |  |
| >50% | 61 (33.5%) | 25 (32.1%) | 36 (34.6%) |  |
| **Glucose_admission (mg/dL)** | 131 [107;190] | 126 [102;184] | 134 [113;196] | 0.063 |
| **HB_admission (g/dL)** | 13.5 (2.03) | 13.7 (1.76) | 13.3 (2.24) | 0.141 |
| **K_admission (mmol/L)** | 4.47 (0.58) | 4.49 (0.60) | 4.44 (0.56) | 0.493 |
| **Na_admission (mmol/L)** | **141 [139;143]** | **141 [140;144]** | **140 [138;142]** | **0.003** |
| **NT-ProBNP_admission (pg/mL)** | 2616 [1178;5314] | 2417 [1025;5834] | 2718 [1534;4770] | 0.304 |
|  |  |  |  |  |
