# Supplementary material for: A New Biomarker Tool for Risk Stratification in “de novo” Acute Heart Failure (OROME)
Source: Front Physiol. 2022 Jan 13;12:736245. doi: 10.3389/fphys.2021.736245 (PMC8793744; doi:10.3389/fphys.2021.736245)
Supplement: Supplementary file 2 [file Table_2.docx]

**Supplementary table 2. Clinical characteristics differences among patients who suffered or not events**

| **Orosomucoid/Omentin (OROME)** | **DownUp** | **Equal** | **UpDown** | **p.overall** |
| --- | --- | --- | --- | --- |
|  | ***N=43*** | ***N=89*** | ***N=22*** |  |
| **Sex (Women)** | **21 (48.8%)** | **27 (30.3%)** | **6 (27.3%)** | **0.080** |
| **Age** | **67.0 [59.5;79.0]** | **71.0 [61.0;78.0]** | **72.5 [62.8;78.0]** | **0.652** |
| **Alcohol:** | **13 (30.2%)** | **35 (40.7%)** | **8 (36.4%)** | **0.509** |
| **Tobacco** | **6 (14.0%)** | **25 (28.7%)** | **4 (18.2%)** | **0.143** |
| **BMI** | **28.9 [27.4;35.1]** | **29.9 [27.1;32.8]** | **28.6 [26.4;34.1]** | **0.987** |
| **Previous peripheral artery** | **2 (4.65%)** | **8 (8.99%)** | **2 (9.09%)** | **0.696** |
| **DM** | **16 (37.2%)** | **35 (39.3%)** | **5 (22.7%)** | **0.347** |
| **Edemas_admission** | **25 (58.1%)** | **43 (48.3%)** | **11 (50.0%)** | **0.566** |
| **COPD** | **1 (2.33%)** | **15 (16.9%)** | **4 (18.2%)** | **0.041** |
| **Hepatomegalia** | **2 (4.76%)** | **9 (12.2%)** | **4 (18.2%)** | **0.236** |
| **HLP:** | **24 (55.8%)** | **50 (56.2%)** | **9 (40.9%)** | **0.418** |
| **HTA** | **34 (79.1%)** | **57 (64.0%)** | **17 (77.3%)** | **0.153** |
| **Previous myocardial infarction** | **5 (11.6%)** | **11 (12.4%)** | **3 (13.6%)** | **1.000** |
| **Previous Stroke** | **3 (6.98%)** | **4 (4.49%)** | **1 (4.55%)** | **0.873** |
| **Heart rate** | **69.7 (14.1)** | **71.3 (13.2)** | **68.4 (12.4)** | **0.628** |
| **Systolic blood preasure_admission** | **144 (28.3)** | **139 (26.4)** | **140 (23.6)** | **0.555** |
| **Salicylic Acid** | **13 (30.2%)** | **28 (32.2%)** | **7 (31.8%)** | **0.975** |
| **Amiodarone** | **4 (9.30%)** | **9 (10.3%)** | **4 (18.2%)** | **0.601** |
| **ARB** | **8 (18.6%)** | **20 (23.0%)** | **3 (13.6%)** | **0.595** |
| **Betablockers** | **38 (88.4%)** | **67 (77.0%)** | **16 (72.7%)** | **0.210** |
| **Digoxin** | **14 (32.6%)** | **18 (20.9%)** | **4 (18.2%)** | **0.274** |
| **Diuretics** | **41 (95.3%)** | **79 (90.8%)** | **22 (100%)** | **0.253** |
| **ACEI** | **31 (72.1%)** | **54 (62.1%)** | **13 (59.1%)** | **0.452** |
| **Ivabradine:** | **1 (2.33%)** | **7 (8.05%)** | **1 (4.55%)** | **0.458** |
| **Metformin:** | **12 (27.9%)** | **28 (32.2%)** | **4 (18.2%)** | **0.426** |
| **Nitrates:** | **3 (6.98%)** | **4 (4.60%)** | **2 (9.09%)** | **0.715** |
| **Creatinine_admission (mg/dL)** | **0.84 [0.70;1.00]** | **1.00 [0.82;1.28]** | **1.21 [0.94;1.30]** | **0.001** |
| **LVEF_admission** |  |  |  | **0.918** |
| **<40%** | **17 (43.6%)** | **41 (51.2%)** | **7 (41.2%)** |  |
| **40-49%** | **8 (20.5%)** | **14 (17.5%)** | **3 (17.6%)** |  |
| **>50%** | **14 (35.9%)** | **25 (31.2%)** | **7 (41.2%)** |  |
| **Glucose_admission (mg/dL)** | **127 [107;192]** | **137 [108;193]** | **132 [111;189]** | **0.919** |
| **HB_admission (g/dL)** | **13.6 (1.91)** | **13.5 (2.08)** | **13.2 (1.80)** | **0.785** |
| **K_admission (mmol/L)** | **4.25 (0.54)** | **4.52 (0.62)** | **4.63 (0.46)** | **0.017** |
| **Na_admission (mmol/L)** | **140 [139;143]** | **140 [138;143]** | **142 [139;144]** | **0.192** |
| **NT-ProBNP_admission** | **2360 [1110;3815]** | **2086 [1126;5261]** | **3431 [1052;5368]** | **0.677** |
