# Supplementary material for: A New Biomarker Tool for Risk Stratification in “de novo” Acute Heart Failure (OROME)
Source: Front Physiol. 2022 Jan 13;12:736245. doi: 10.3389/fphys.2021.736245 (PMC8793744; doi:10.3389/fphys.2021.736245)
Supplement: Supplementary file 3 [file Table_3.docx]

**Supplementary table 3.** Variable selected with respect to three methods: LassoAIC, step-by-step with validation, step-by-step selection with validation by randomization, and step-by-step selection with validation using 200 bootstrap samples (Selection% column in the tables). The number shown in this column is the percentage of times (with respect to the 200 bootstrap samples) that this variable is selected.

| Model 1 |  |  |  | Model 1B |  |  |  |  |
| --- | --- | --- | --- | --- | --- | --- | --- | --- |
| Var | % Selection (B=200 | Back | LassoAIC |  | Var | Selection % (B=200) | Back | LassoAIC |
| Tabaco | 0.1959 |  |  |  | Tabaco | 0.2653 |  |  |
| HR | 0.2160 |  |  |  | Alcohol | 0.2806 |  | x |
| Alcohol | 0.2512 |  |  |  | Na | 0.3112 |  |  |
| Sex | 0.2613 |  |  |  | Hb | 0.4234 |  | x |
| Na | 0.3015 |  |  |  | HR | 0.4337 |  | x |
| Orosomucoid | 0.3266 |  |  |  | Sex | 0.4490 |  | x |
| Glucose | 0.3718 |  |  |  | DP | 0.5510 |  | x |
| Age | 0.4221 |  |  |  | Glucose | 0.5714 |  | x |
| Creatinine | 0.4372 |  |  |  | Age | 0.6071 | x | x |
| Omentin | 0.4573 | x | x |  | Omentin | 0.6275 |  | x |
| Hb | 0.5226 |  |  |  | BMI | 0.6530 | x | x |
| BMI | 0.5578 | x | x |  | LVEF | 0.7143 | x | x |
| DP | 0.6130 |  |  |  | Score_drug | 0.7143 | x | x |
| Score_drug | 0.7839 | x | x |  | Creatinine | 0.7857 | x | x |
| K | 0.9396 | x | x |  | Score | 0.9234 | x | x |
| Score | 0.9447 | x | x |  | K | 1 | x | x |
| LVEF | 0.9648 | x | x |  |  |  |  |  |
| NT-proBNP | 1 | x | x |  |  |  |  |  |
|  |  |  |  |  |  |  |  |  |
|  |  |  |  |  |  |  |  |  |
|  |  |  |  |  |  |  |  |  |
| Model 1C |  |  |  | Model 1D |  |  |  |  |
| Var | % Selection (B=200 | Back | LassoAIC |  | Var | Selection % (B=200) | Back | LassoAIC |
|  |  |  |  |  |  |  |  |  |
| Tabaco | 0.1878 |  |  |  | Tabaco | 0.2110 |  | x |
| Orosomucoid | 0.2132 |  |  |  | Orosomucoid | 0.2311 | x | x |
| Na | 0.2893 |  |  |  | BMI | 0.2764 |  |  |
| Sex | 0.3147 |  |  |  | Na | 0.3367 |  | x |
| HR | 0.3147 |  |  |  | Alcohol | 0.3467 |  | x |
| Alcohol | 0.3248 |  |  |  | DP | 0.4120 |  |  |
| Hb | 0.3705 |  |  |  | Hb | 0.4221 |  | x |
| DP | 0.4568 |  |  |  | Sex | 0.4422 |  | x |
| BMI | 0.5228 | x | x |  | Age | 0.4874 | x | x |
| Glucose | 0.5939 |  | x |  | HR | 0.5075 |  | x |
| LVEF | 0.6091 | x | x |  | Glucose | 0.5427 |  | x |
| Age | 0.6751 | x | x |  | Omentin | 0.6482 |  | x |
| Score_drug | 0.6852 | x | x |  | LVEF | 0.7136 | x | x |
| Creatinine | 0.7817 | x | x |  | Score_drug | 0..7186 | x | x |
| K | 0.9796 | x | x |  | Creatinine | 0.8894 | x | x |
| Score | 1 | x | x |  | K | 0.9648 | x | x |
|  |  |  |  |  | Score | 1 | x | x |
|  |  |  |  |  |  |  |  |  |
|  |  |  |  |  |  |  |  |  |
|  |  |  |  |  |  |  |  |  |
| Model 2 |  |  |  | **Model 3** |  |  |  |  |
| Var | % Selection (B=200 | Back | LassoAIC |  | Var | Selection % (B=200) | Back | LassoAIC |
|  |  |  |  |  |  |  |  |  |
| Alcohol | 0.2538 |  | x |  | Tabaco | 0.2653 |  |  |
| HR | 0.2690 |  | x |  | Alcohol | 0.2806 |  | x |
| Tabaco | 0.3045 |  | x |  | Na | 0.3112 |  |  |
| Hb | 0.3147 |  | x |  | Hb | 0.4234 |  |  |
| Na | 0.3197 |  | x |  | HR | 0.4337 |  | x |
| Sex | 0.3350 |  |  |  | Sex | 0.4489 |  | x |
| DP | 0.3654 |  | x |  | DP | 0.5510 |  | x |
| Score | 0.5177 | x | x |  | Glucose | 0.5714 |  | x |
| Score*Oroso | 0.5177 |  | x |  | Age | 0.6071 | x | x |
| BMI | 0.5888 | x | x |  | Omentin | 0.6275 |  | x |
| Age | 0.6802 | x | x |  | BMI | 0.6530 | x | x |
| LVEF | 0.6802 | x | x |  | LVEF | 0.7142 | x | x |
| Glucose | 0.6954 |  | x |  | Score_drug | 0.7142 | x | x |
| Creatinine | 0.7563 | x | x |  | Creatinine | 0.7857 | x | x |
| Orosomucoid | 0.8426 | x | x |  | Score | 0.9234 | x | x |
| K | 0.9390 | x | x |  | K | 1 | x | x |
| Score_drug*Oroso | 0.9746 | x | x |  |  |  |  |  |
| Score_drug | 1 | x | x |  |  |  |  |  |
|  |  |  |  |  |  |  |  |  |
|  |  |  |  |  |  |  |  |  |
|  |  |  |  |  |  |  |  |  |
|  |  |  |  |  |  |  |  |  |
| Model 4A |  |  |  | M**odel 4B** |  |  |  |  |
| Var | % Selection (B=200 | Back | LassoAIC |  | Var | Selection % (B=200) | Back | LassoAIC |
|  |  |  |  |  |  |  |  |  |
| Alcohol | 0.2436 |  | x |  | Alcohol | 0.1809 |  |  |
| Tabaco | 0.2690 |  | x |  | DP | 0.2763 |  | x |
| HR | 0.3045 |  | x |  | Sex | 0.3015 |  |  |
| Sex | 0.3299 |  | x |  | Na | 0.3065 |  | x |
| Na | 0.3350 |  | x |  | Tabaco | 0.3216 |  | x |
| Hb | 0.3502 |  | x |  | Hb | 0.3618 |  | x |
| DP | 0.3857 |  | x |  | HR | 0.3718 |  | x |
| Score*Oroso | 0.4517 |  | x |  | Omentin | 0.4422 | x | x |
| Omentin | 0.4822 |  | x |  | Score*Oroso | 0.4422 |  | x |
| Age | 0.5532 | x | x |  | BMI | 0.4874 | x | x |
| BMI | 0.5634 | x | x |  | Age | 0.5427 | x | x |
| Score | 0.6142 | x | x |  | Score*Omentin | 0.5678 | x | x |
| Glucose | 0.6192 |  | x |  | Glucose | 0.6080 |  | x |
| LVEF | 0.6802 | x | x |  | Score | 0.6783 |  | x |
| Creatinine | 0.7512 | x | x |  | LVEF | 0.6934 | x | x |
| Orosomucoid | 0.7969 | x | x |  | Creatinine | 0.7939 | x | x |
| K | 0.9492 | x | x |  | Orosomucoid | 0.8542 | x | x |
| Score_drug*Oroso | 0.9644 | x | x |  | K | 0.9497 | x | x |
| Score_drug | 1 | x | x |  | Score_drug*Oroso | 0.9798 | x | x |
|  |  |  |  |  | Score_drug | 1 | x | x |
|  |  |  |  |  |  |  |  |  |
|  |  |  |  |  |  |  |  |  |
|  |  |  |  |  |  |  |  |  |
|  |  |  |  |  |  |  |  |  |
|  |  |  |  |  |  |  |  |  |
| Model 5A |  |  |  | Model 5B |  |  |  |  |
| Var | % Selection (B=200) | Back | LassoAIC |  | Var | Selection % (B=200) | Back | LassoAIC |
|  |  |  |  |  |  |  |  |  |
| HR | 0.2385 |  | x |  | Age | 0.2052 |  | x |
| Tabaco | 0.2588 |  |  |  | Sex | 0.2105 |  |  |
| DP | 0.2639 |  | x |  | DP | 0.2894 |  |  |
| Age | 0.2791 |  | x |  | Tabaco | 0.3105 |  |  |
| Alcohol | 0.2944 |  | x |  | BMI | 0.3105 | x | x |
| Sex | 0.3147 |  |  |  | Na | 0.3105 |  |  |
| Na | 0.3350 | x |  |  | Score_drug*Oroso | 0.3157 |  | x |
| Omentin | 0.3451 | x | x |  | Alcohol | 0.3421 |  | x |
| BMI | 0.4213 |  | x |  | HR | 0.3842 |  | x |
| Score*Oroso | 0.4517 |  | x |  | Omentin | 0.4210 | x | x |
| Creatinine | 0.4619 |  |  |  | Score*Omentin | 0.4526 | x |  |
| Hb | 0.5228 |  | x |  | Creatinine | 0.5263 |  | x |
| Glucose | 0.5583 | x | x |  | Glucose | 0.5315 |  | x |
| Score | 0.6497 | x | x |  | Hb | 0.5894 |  | x |
| K | 0.9035 | x | x |  | Score | 0.6473 | x |  |
| Orosomucoid | 0.9187 | x |  |  | K | 0.8421 | x | x |
| Score_drug*Oroso | 0.9644 | x | x |  | Orosomucoid | 0.9105 | x |  |
| LVEF | 0.9796 | x | x |  | Score_drug*Oroso | 0.9263 | x | x |
| Score_drug | 0.9847 | x | x |  | LVEF | 0.9736 | x | x |
| NT-proBNP | 1 | x | x |  | Score_drug | 0.9842 | x | x |
|  |  |  |  |  | NT-proBNP | 1 | x | x |
