# Supplementary figures and images for: A New Biomarker Tool for Risk Stratification in “de novo” Acute Heart Failure (OROME)
Source: Front Physiol. 2022 Jan 13;12:736245. doi: 10.3389/fphys.2021.736245 (PMC8793744; doi:10.3389/fphys.2021.736245)

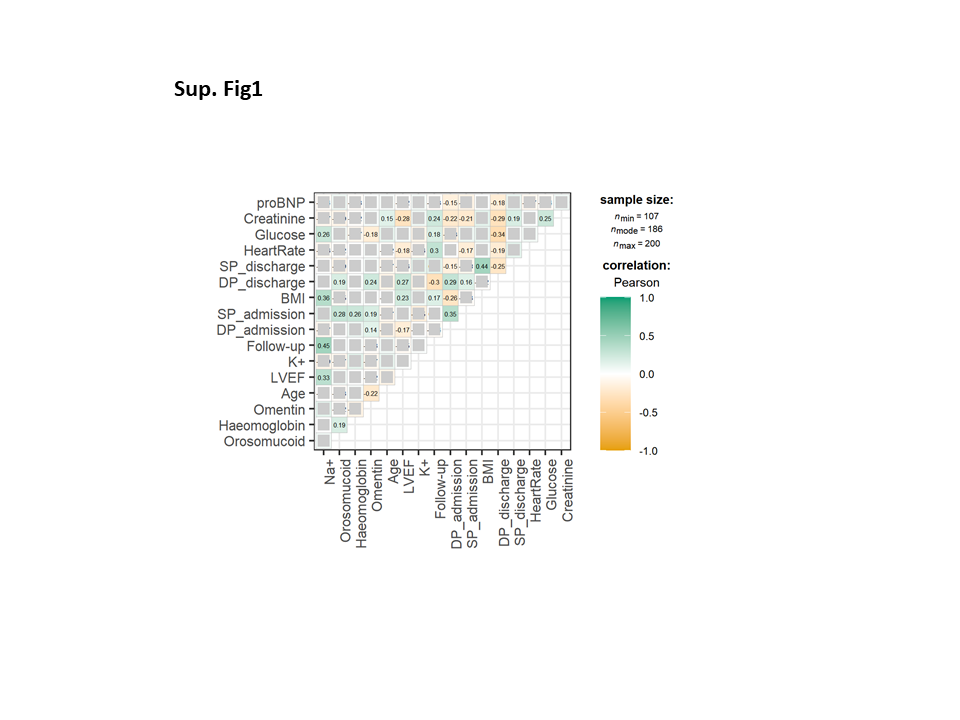

Supplement: Supplementary file 4 [file Image_1.tif]

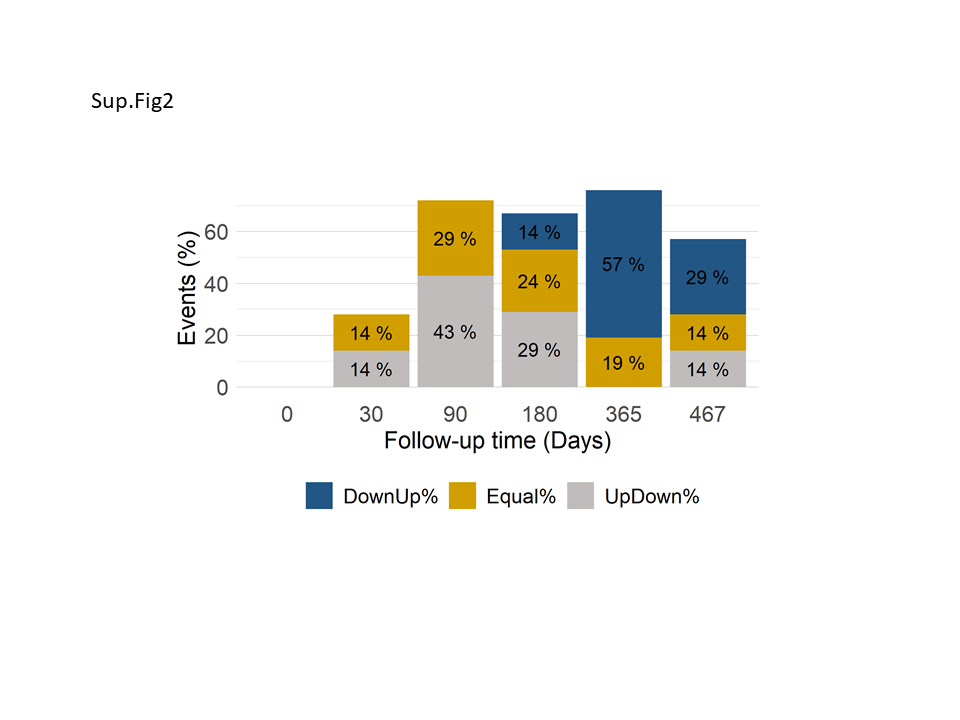

Supplement: Supplementary file 5 [file Image_2.tif]

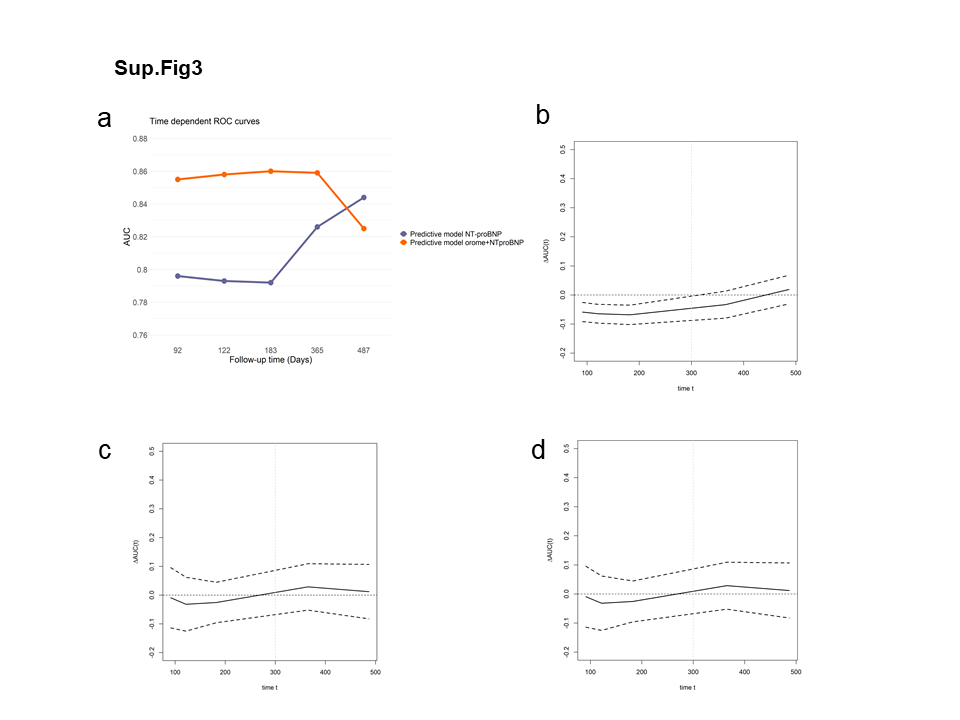

Supplement: Supplementary file 6 [file Image_3.tif]

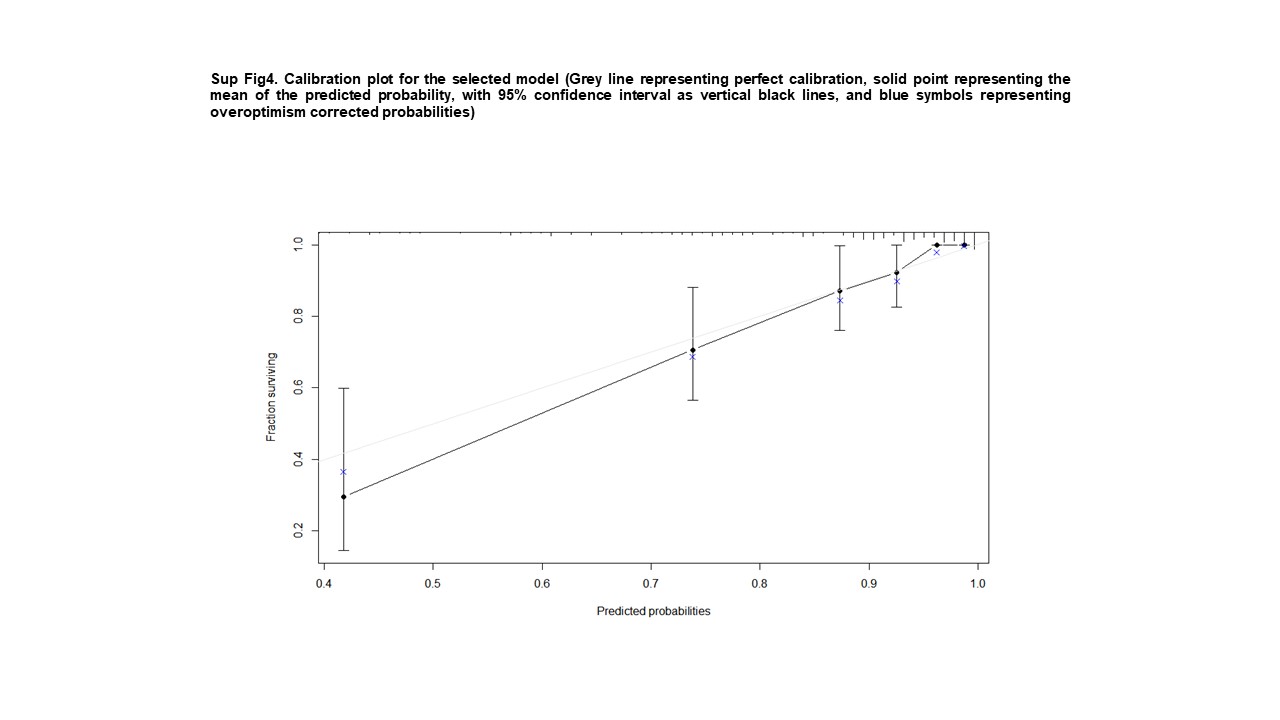

Supplement: Supplementary file 7 [file Image_4.JPEG]
